# Assessment of Incidence of and Surveillance Burden for Hepatocellular Carcinoma Among Patients With Hepatitis C in the Era of Direct-Acting Antiviral Agents

**DOI:** 10.1001/jamanetworkopen.2020.21173

**Published:** 2020-11-18

**Authors:** Qiushi Chen, Turgay Ayer, Madeline G. Adee, Xiaojie Wang, Fasiha Kanwal, Jagpreet Chhatwal

**Affiliations:** 1 Institute for Technology Assessment, Massachusetts General Hospital, Harvard Medical School, Boston; 2 Harold and Inge Marcus Department of Industrial and Manufacturing Engineering, Pennsylvania State University, University Park; 3 H. Milton Stewart School of Industrial and Systems Engineering, Georgia Institute of Technology, Atlanta; 4 Department of Medicine, Baylor College of Medicine, Houston, Texas; 5 Houston Veterans Affairs Health Services Research and Development Center of Excellence, Michael E. DeBakey Veterans Affairs Medical Center, Houston, Texas

## Abstract

**Importance:**

In the US, hepatocellular carcinoma (HCC), primarily associated with hepatitis C virus (HCV) infection, is the fastest rising cause of cancer-related death. Wider use of highly effective direct-acting antiviral agents (DAAs) substantially reduces the burden of chronic HCV infection, but the subsequent impacts with HCV-associated HCC remain unknown.

**Objective:**

To assess projected changes in the incidence rate of and surveillance burden for HCC in the era of DAA treatment for HCV.

**Design, Setting, and Participants:**

This decision analytical model study was performed from January 2019 to February 2020, using an individual-level state-transition simulation model to simulate disease progression, screening, and different waves of antiviral treatments for HCV in the US from 2012 to 2040.

**Interventions:**

Current clinical management for chronic HCV infection.

**Main Outcomes and Measures:**

Model outcomes were projected temporal trends and age distribution of incident HCC cases and candidates for HCC surveillance among patients with viremia and patients with virologically cured HCV.

**Results:**

The simulation model projected that the annual incidence of HCC among patients with viremia and patients with virologically cured HCV will continue increasing to 24 000 (95% uncertainty interval [UI], 18 000-31 000) cases until 2021. In patients with virologically cured HCV, incident HCC cases are projected to increase from 1000 (95% UI, 500-2100) in 2012 to the peak of 7000 (95% UI, 5000-9600) in 2031 with a subsequent decrease to 6000 (95% UI, 4300-8300) by 2040. The proportion of incident HCC cases that occur in individuals with virologically cured HCV is estimated to increase from 5.3% in 2012 to 45.8% in 2040. The number of candidates for HCC surveillance in the population with virologically cured HCV is projected to increase from 106 000 (95% UI, 70 000-178 000) in 2012 to the peak of 649 000 (95% UI, 512 000-824 000) in 2030 and decrease to 539 000 (95% UI, 421 000-687 000) by 2040, while the proportion of all candidates for surveillance who are virologically cured is estimated to increase from 8.5% to 64.6% during the same period. The average age of HCC incidence and surveillance candidates is estimated to increase from 55 in 2012 to 72 and 71, respectively, by 2040.

**Conclusions and Relevance:**

The results of this study suggest that the burden of HCC will shift from patients with viremia to patients with virologically cured HCV, and to older populations. Appropriate management may be warranted for early detection of HCC in patients who may no longer be receiving specialty care for liver conditions.

## Key Points

**Question:**

What are the projected changes in incidence of and surveillance burden for hepatocellular carcinoma (HCC) among populations with hepatitis C virus (HCV) in the era of direct-acting antiviral agents (DAAs)?

**Findings:**

In this decision-analytical model, the incidence of HCC associated with HCV and candidates for HCC surveillance were projected to increase before starting to decrease in the era of DAAs. The burden of HCC associated with HCV was projected to shift from patients with viremia to individuals with virologically cured HCV and to older individuals.

**Meaning:**

Results of this study suggest that routine HCC surveillance is needed for early detection of HCC in individuals with virologically cured hepatitis C who may no longer be receiving specialty care of liver diseases.

## Teaser

This decision analytical model study assesses the burden of hepatocellular carcinoma surveillance in the era of new hepatitis C antiviral treatments by projecting the number and the characteristics of new hepatocellular carcinoma incident cases and candidates for routine hepatocellular carcinoma surveillance.

## Introduction

Hepatocellular carcinoma (HCC) is the fastest rising cause of cancer-related mortality in the US.^1,2^ The incidence of HCC has increased over the last 2 decades owing to the hepatitis C virus (HCV) epidemic.^3^ Most HCC cases are diagnosed in advanced stages, with a median survival less than 1 year.^4^ Regular surveillance for HCC may help improve early cancer detection rates when curative treatment can be applied and is recommended in patients with HCV-associated cirrhosis.^5,6,7^

The availability of direct-acting antiviral agents (DAAs) for HCV treatment has substantially altered the landscape of HCV. Though new DAA regimens can result in a virological cure (ie, sustained virological response [SVR]) in more than 90% of patients with HCV,^8^ many patients remain at risk of developing HCC after virological cure.^9,10^ Current clinical guidelines recommend that patients with virologically cured HCV with cirrhosis (or advanced fibrosis) to undergo routine HCC surveillance every 6 months.^11,12^

Though the incidence rate of HCC in patients with virologically cured HCV is lower than that in patients with viremia, the absolute number of patients with virologically cured HCV requiring routine HCC surveillance is estimated to increase in the next decade.^13^ Patients with virologically cured HCV may experience improvement in liver function and hence live longer and could undergo surveillance for several years. In contrast, most patients with virologically cured HCV, despite being at risk for HCC, may not undergo routine HCC surveillance because of the perceived lower risk. Previous studies have reported that HCC surveillance rates are much lower in patients receiving primary care vs patients receiving specialty care.^14,15^ Thus, identifying HCC cases through surveillance in this cohort could be more challenging than that in other individuals at risk.

Estimating the burden of HCC surveillance in the cohorts of patients with virologically cured HCV may help guide primary and specialty care providers. The objective of this study was to evaluate the burden of HCC surveillance in the era of new HCV antiviral treatments by projecting the number and the characteristics of new HCC incident cases and candidates for routine HCC surveillance.

## Methods

We used a previously developed mathematical model, Hepatitis C Disease Burden Simulation model (HEP-SIM),^8,16^ to simulate the population with HCV who would be considered candidates for HCC surveillance in the era of DAAs in the US. The HEP-SIM is an individual-level state-transition model that simulates the changing landscape of HCV by replicating disease progression, screening for HCV, different waves of antiviral treatments for HCV, and insurance coverage from 2001 to 2040. The model was previously validated with results of the National Health and Nutrition Examination Survey (NHANES) 2003-2010,^17^ reports from the Centers for Disease Control and Prevention,^18^ and natural history results from a long-term follow-up study of patients with advanced fibrosis.^19^ In this study, we projected temporal trends in HCC incidence and the changing characteristics of the number of patients with HCV (patients with viremia and patients with virologically cured HCV) who will be candidates for routine HCC surveillance in the era of DAAs. All data used in this analysis were publicly available and this study thus did not require approval from an institutional review board. This study followed the Consolidated Health Economic Evaluation Reporting Standards (CHEERS) reporting guideline for decision analytical model studies. We describe the major model components; further model details can be found elsewhere.^16^

### Population Characteristics and Natural History

The population in the HEP-SIM model represented patients with HCV in the US. The population included the NHANES population and the non-NHANES population, including incarcerated (in prisons), homeless, active-duty military, and nursing home populations. Patient HCV genotypes, liver disease states, awareness rate, and age distribution were estimated from published studies and other sources (eTable 1 and eTable 2 in the Supplement). New incidence of HCV infections was based on the Centers for Disease Control and Prevention–reported estimates for the years 2006 to 2015 (eTable 3 in the Supplement), and we assumed that the annual incidence would change at the average rate observed during 2006 to 2015 until 2026 and then become constant.

We simulated the natural history of HCV, which was defined by different stages of liver disease, with chronic HCV infection defined using METAVIR fibrosis scores (F0, no fibrosis; F1, portal fibrosis without septa; F3, numerous septa without fibrosis; and F4, cirrhosis), decompensated cirrhosis, HCC, liver transplant, and liver-related death. Rates of fibrosis progression and decompensation were estimated from published metaregression analysis and observational studies^20,21,22^ (eTable 4 in the Supplement). Patients with decompensated cirrhosis or with HCC had higher mortality rates^23^ and were eligible to for liver transplant.^24,25,26^

### Screening and Treatment for Patients With HCV in the DAA Era

To accurately represent the HCV disease burden that would subsequently affect the burden of HCC, we simulated the clinical management of HCV, ie, screening and treatment, from 2001 to 2040. In each year, patients with HCV could be diagnosed through usual care, which included risk-based screening (eTable 5 in the Supplement). Since 2013, we implemented one-time birth-cohort screening for individuals born between 1945 and 1965 among the NHANES, nursing home, and homeless populations in addition to the diagnosis through usual care. From 2020 to projections to 2040, we included one-time universal screening of all adults between aged 18 to 79 years, following the updated screening recommendation by the US Preventive Services Task Force.^27^ For universal screening, we assumed the screening rate of 9% per year, which is the rate observed in the one-time birth-cohort screening policy.^28,29^

For HCV treatment, we modeled multiple waves of antiviral treatments available in the US during different periods.^16^ The market share of different types of treatment regimens for each HCV genotype was estimated using commercial claims from research organizations (QuintilesIMS and Ipsos) (eTable 6 in the Supplement). Treatment efficacy measured as SVR rate was dependent on the regimen type, individual HCV genotype, presence of cirrhosis, and failure of previous treatment if any, which were estimated based on data from multiple clinical trials and real-world studies (eTable 7 in the Supplement). For all populations except individuals who were incarcerated or homeless, the number of patients receiving treatment in each year was estimated based on published data and drug sales until 2016 (eTable 8 in the Supplement). In the individuals who were incarcerated and homeless, treatment uptake was 1% per year from 2010 to 2016 and 5% per year starting in 2017.^30^ Our model did not consider treatment for patients with decompensated cirrhosis. With limited treatment capacity, priorities were given to patients with bridging fibrosis (F3) or compensated cirrhosis. Patients whose previous treatment failed may be eligible for retreatment, depending on his previous regimen, new available regimen, and presence of cirrhosis (eTable 7 in the Supplement).^16^

### HCC Incidence

Patients with HCV having bridging fibrosis (F3), compensated, and decompensated cirrhosis were at risks of developing HCC.^9,10,22^ The HCC risk in patients with virologically cured HCV was lower than in patients with viremia (eTable 4 in the Supplement).^9^ Patients with compensated cirrhosis could progress to decompensated cirrhosis even after achieving virological cure of HCV and they remained at risk of developing HCC.^31^ We assumed a lower risk of HCC in patients with decompensated cirrhosis with virologically cured HCV than in those with viremia based on a prospective cohort study.^32^

### Subpopulations

We considered different subpopulations in the HEP-SIM model (eTable 9 in the Supplement) based primarily on their health insurance status. We assumed that for the NHANES and nursing home populations, all patients aged 65 years and older were covered by Medicare, and patients aged less than 65 years were covered by private (49.8%), Medicaid (9.2%), or other public insurance (14.3%), or were uninsured (26.7%) based on NHANES data.^13,28,33^ For the population of individuals who were homeless, the insurance distribution was estimated from a 2012 study (3.2% privately insured, 31.5% Medicaid, 65.3% uninsured).^34^ We incorporated insurance expansion during the period from 2014 to 2017 owing to the implementation of the Affordable Care Act (eTable 10 in the Supplement). We also include patients who were incarcerated as a separate population because this population represents a substantial group of viremic infections that were not accounted for in NHANES data.^35^

### Model Outcomes

Our primary model outcome was the number of patients who developed HCC and the number of patients who were candidates for routine HCC surveillance from 2012 to 2040. Candidates for HCC surveillance were defined as patients with F3 fibrosis stage, or compensated cirrhosis, with or without virological cure. In addition, we assumed that 15% (range, 5%-25%) of patients with compensated cirrhosis (eligible for liver transplant) were candidates for HCC surveillance.^36,37^ We further stratified the number of candidates for HCC surveillance by viremia status (ie, with viremia or virologically cured HCV), cirrhosis, and subpopulation category. We also projected the age distribution of HCC incident cases and surveillance candidates for each year. To account for model uncertainty in parameters, we conducted probabilistic sensitivity analysis by sampling model inputs from the recommended statistical distributions^38^ (eTable 11 in the Supplement). We evaluated model outputs for sampled parameters by 2000 iterations and presented the 95% uncertainty intervals (UIs) for model outcomes. The simulation model was developed in C++ programming language and all statistical analysis was performed in R version 3.6.0 (R Project for Statistical Computing).

## Results

### HCC Incidence

**Figure 1.**
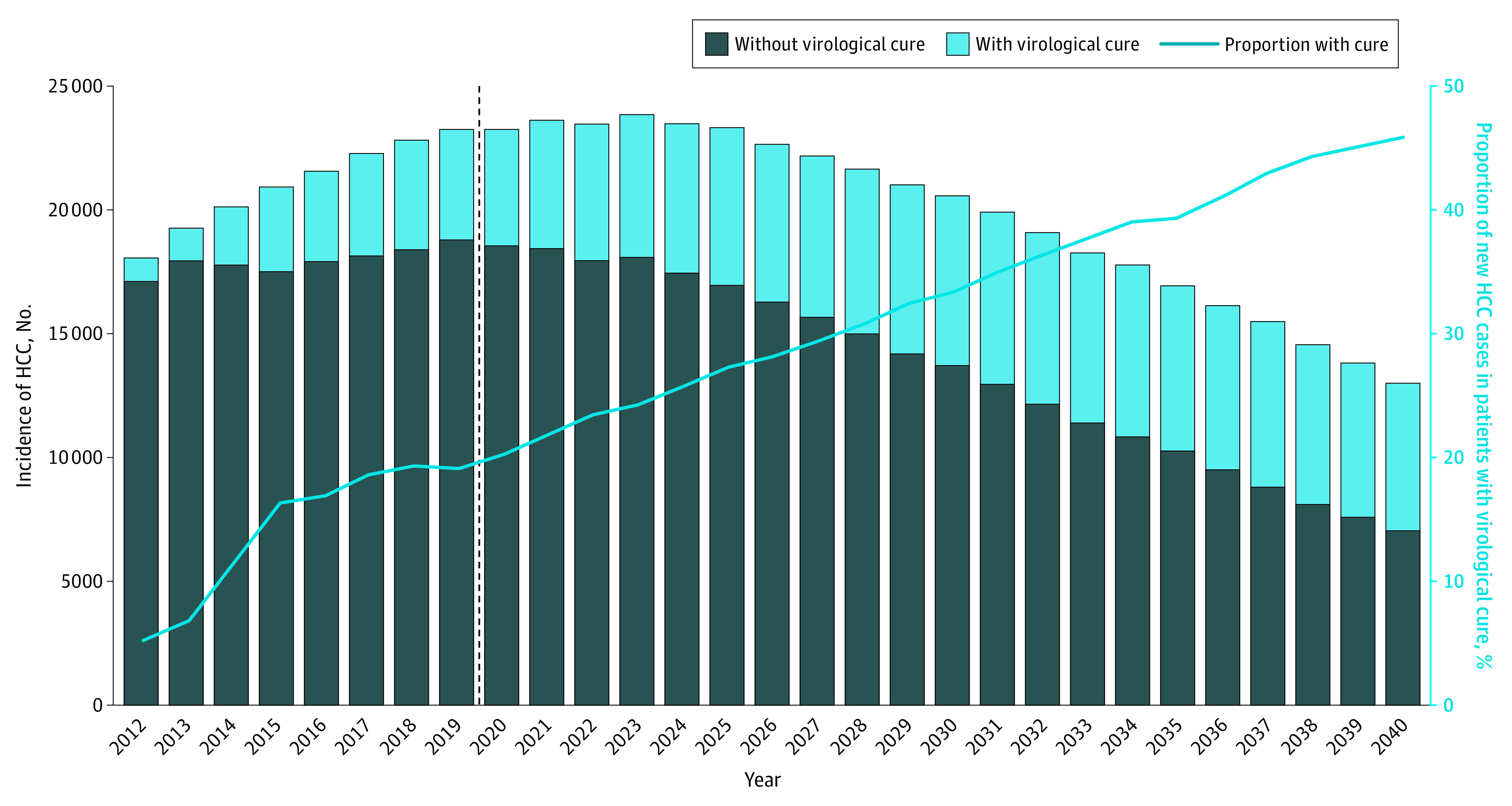
Projection of Annual Hepatocellular Carcinoma (HCC) Incidence by Cure Status From 2012 to 2040 The dashed line represents the starting year of the universal hepatitis C virus screening in US adults.

This decision analytical model study was conducted from January 2019 to February 2020. In 2012, the annual incidence of HCC among patients with HCV (with viremia and virologically cured HCV) was 18 000 (95% UI, 11 000-32 000). The annual incidence of HCV-associated HCC is projected to increase to 24 000 (95% UI, 18 000-31 000) cases in 2021 and decrease to 13 000 (95% UI, 11 000-16 000) cases by 2040 (Figure 1). In 2012, 1000 (95% UI, 500-2100) new HCC cases (5.3% of all new cases) developed in patients with virologically cured HCV; this number is projected to peak at 7000 (95% UI, 5000-9600) new cases (35.0% of all new cases) in 2031 and then decrease to 6000 (95% UI, 4300-8300) new cases (45.8% of all new cases) by 2040. Most of the HCC cases would develop in patients with cirrhosis. The number of new HCC cases among patients with cirrhosis who achieved virological cure increased substantially from 700 (95% UI, 300-1700) in 2012 to 4500 (95% UI, 3000-6700) in 2040 (eFigure 1 in the Supplement).

We estimated that between 2012 and 2040, the cumulative incidence of HCV-associated HCC would be 583 000 cases (95% UI, 458 000 cases to 738 000 cases), and 27.1% (95% UI, 20.3%-34.7%) of these cases would develop among patients with virologically cured HCV. The 95% UIs for the annual HCC incidence by virologically cured status in each year are presented in eFigure 2 in the Supplement.

### HCC Surveillance Candidates

**Figure 2.**
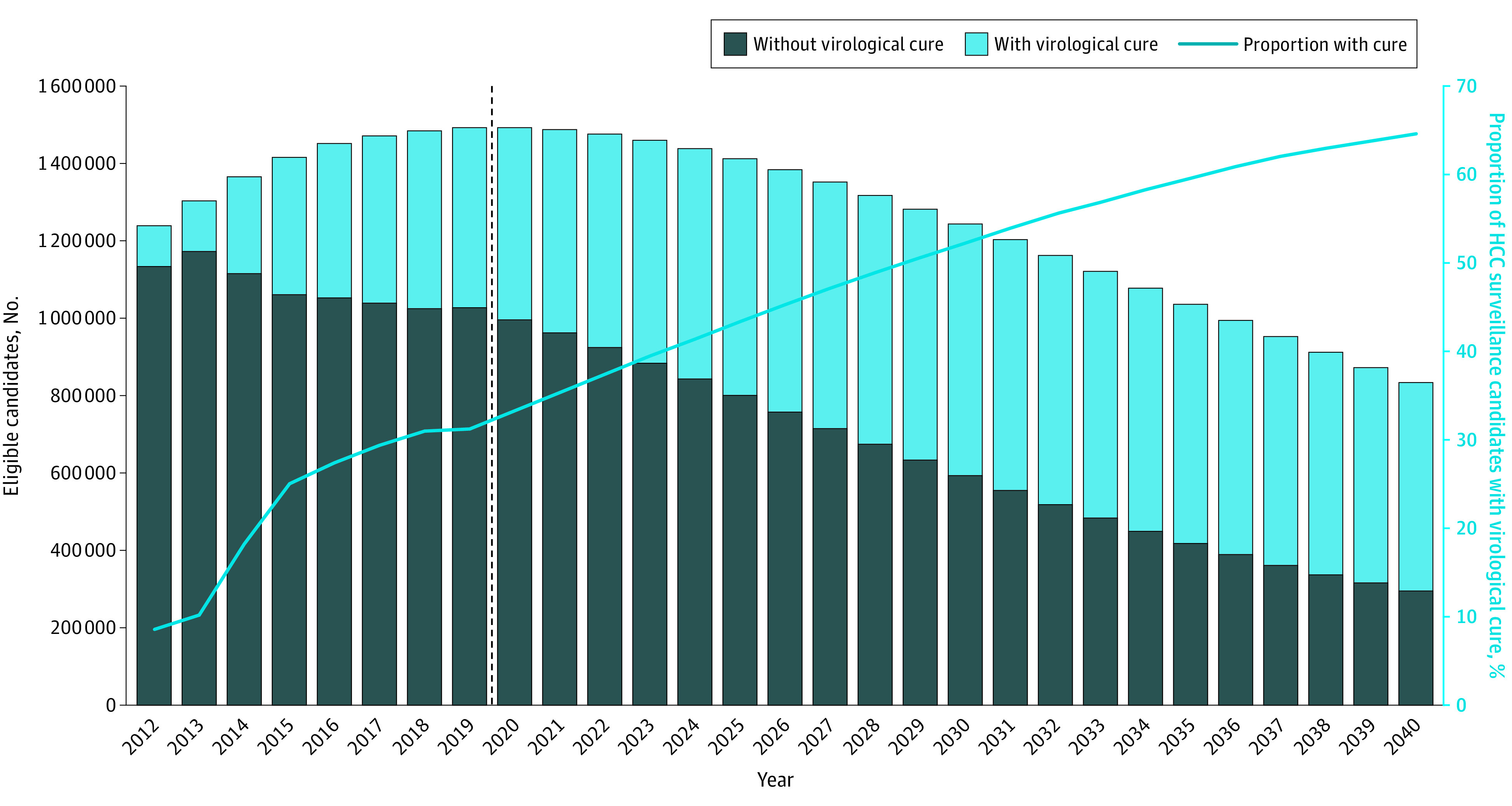
Projection of Number of Candidates for Hepatocellular Carcinoma (HCC) Surveillance by Cure Status From 2012 to 2040 The dashed line represents the starting year of the universal hepatitis C virus screening in US adults.

Our model projected that in 2012, a total of 1.24 million (95% UI, 0.96 million -1.71 million) patients with HCV (with viremia and virologically cured HCV) were at risk of developing HCC and would be candidates for routine HCC surveillance in the US. The number of candidates eligible for HCC surveillance is estimated to increase to 1.49 million (95% UI, 1.23 million to 1.85 million) in 2020 and then gradually decrease to 0.83 million (95% UI, 0.66 million to 1.04 million) by the end of 2040 (Figure 2). In 2012, 106 000 (95% UI, 70 000-178 000) surveillance candidates were those who had achieved virological cure; this number is projected to peak at 649 000 (95% UI, 512 000-824 000) in 2030 and then decrease to 539 000 (95% UI, 421 000-687 000) individuals by 2040. However, the proportion of all HCC surveillance candidates who are virologically cured is estimated to increase from 8.5% in 2012 to 64.6% in 2040. We also projected that the proportion of surveillance candidates with cirrhosis (compensated and decompensated) is estimated to increase from 42.8% in 2012 to 49.6% in 2040, and that the proportion of patients with cirrhosis among patients with virologically cured HCV is estimated to increase from 34.5% to 43.5% in the same time period (eFigure 3 in the Supplement).

**Figure 3.**
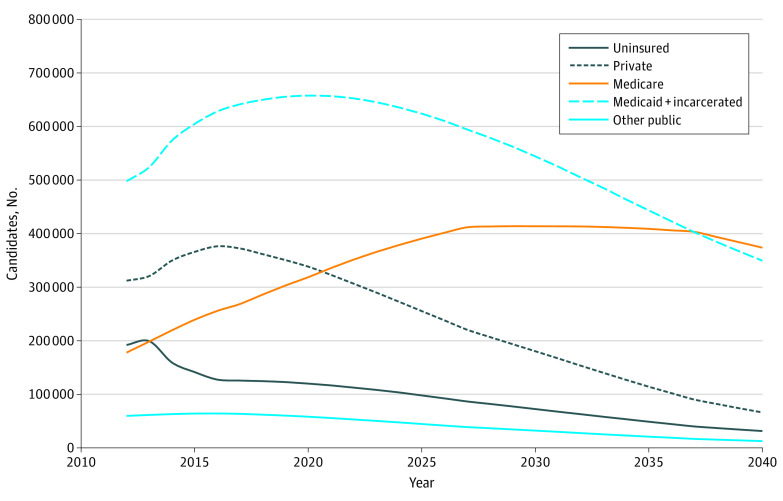
Number of Candidates for Hepatocellular Carcinoma Surveillance by Insurance Subgroup From 2012 to 2040

We further analyzed HCC surveillance candidates by subpopulation (Figure 3). In 2012, 312 000 (95% UI, 225 000-443 000) patients (25.2%) who were candidates for HCC surveillance were covered by private insurance, 178 000 (95% UI, 111 000-274 000) patients (14.4%) by Medicare, 498 000 (95% UI, 390 000-679 000) patients (40.2%) and by state-administered programs (including Medicaid and the population incarcerated in state prisons), 60 000 (95% UI, 44 000-85 000) patients (4.8%) by other public insurance, and 192 000 (95% UI, 144 000-265 000) patients (15.5%) were uninsured. The number of HCC surveillance candidates increased in the privately insured pool to 376 000 (95% UI, 277000-509000) patients (25.9%) by 2016 and decreased in the uninsured pool to 127 000 (95% UI, 96 000-168 000) patients (8.8%) because of the implementation of the Affordable Care Act in 2014. The number of HCC surveillance candidates covered by Medicare increased further because of the aging population of patients with HCV (with viremia and virologically cured HCV) and is expected to surpass the number of candidates who are privately insured from 2021 to 2040 (Figure 3). Among all payers, the state-administered programs (Medicaid and state prisons) is estimated to have the highest burden of HCC surveillance: the number of HCC surveillance candidates incarcerated in state prisons or covered by Medicaid is projected to peak at 658 000 (95% UI, 557 000-786 000) in 2020 and decrease to 349 000 (95% UI, 280 000-427 000) by the end of 2040.

### Aging of HCC Population

**Figure 4.**
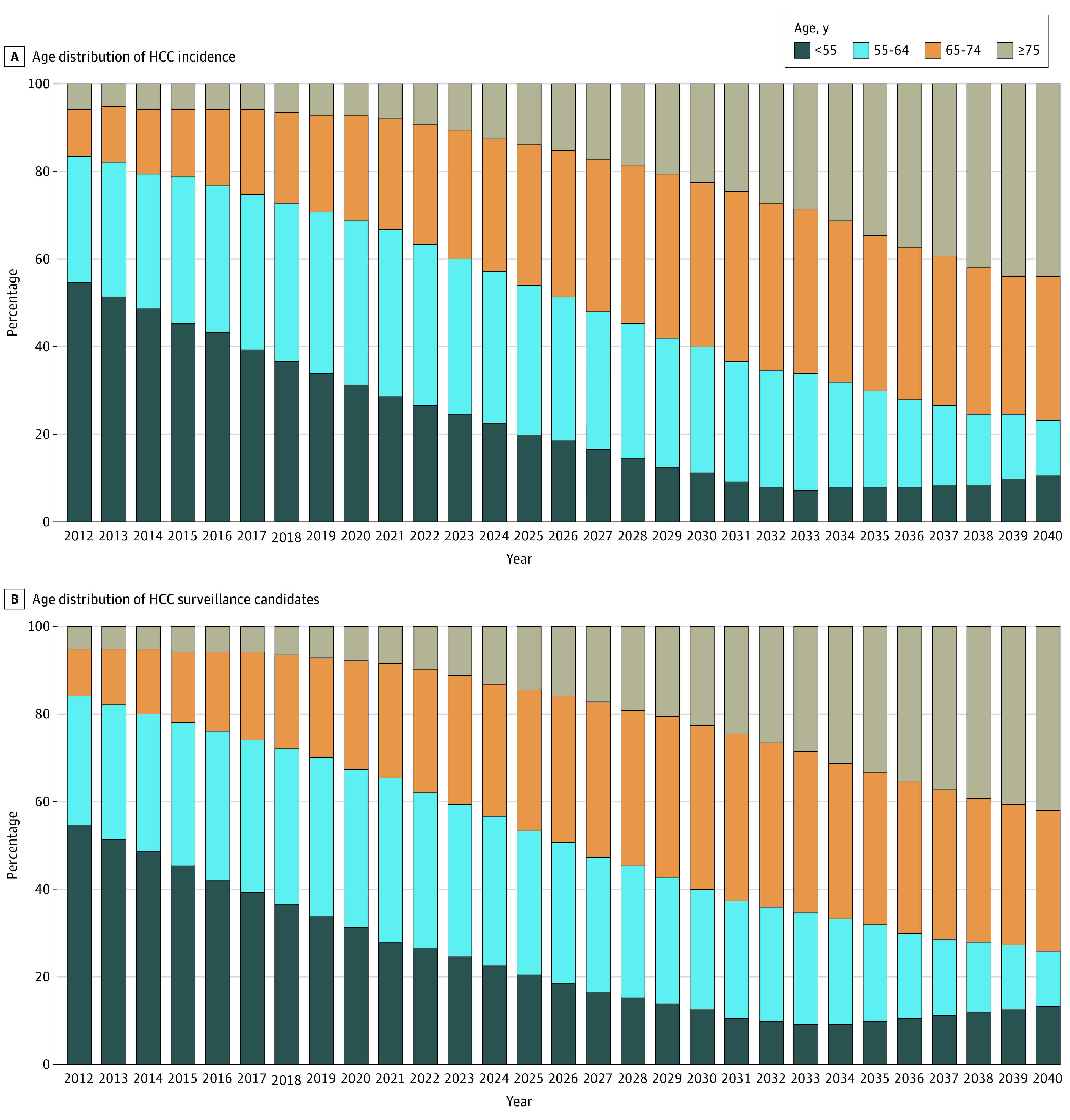
Age Distribution of HCC Incidence and HCC Surveillance Candidates From 2012 to 2040

The average age of HCC incidence is projected to increase from 55 in 2012 and to 72 in 2040 (eFigure 4 in the Supplement). The proportion of incident HCC cases older than age of 65 years is projected to increase from 16.4% in 2012 to 76.5% in 2040 (Figure 4). Similarly, the average age of surveillance candidates is projected to increase from 55 years in 2012 to 71 years in 2040. The proportion of surveillance candidates aged 65 years and older is projected to increase from 16.1% in 2012 to 73.8% in 2040.

## Discussion

The incidence of HCC has increased over the last 2 decades primarily because of the chronic HCV.^3^ With the availability and wider use of new DAAs for HCV treatment, the burden of HCC and routine HCC surveillance attributable to HCV could change in the era of DAAs. In this modeling study, we found that the incidence of HCC is estimated to continue to increase until 2021. Between 2012 and 2040, 583 000 patients with HCV (with viremia and virologically cured HCV) were estimated to develop HCC, and 27% of those cases would be among patients with virologically cured HCV. The number of candidates eligible for HCC surveillance is estimated to increase from 1.24 million in 2012 to 1.49 million in 2020, while the burden of surveillance is expected to shift from patients with viremia to individuals who achieved virological cure. In 2012, 9% of all candidates for HCV surveillance were virologically cured, which is projected to increase to 65% by 2040. The average age of HCC incidence and surveillance candidates is also projected to shift from 55 years in 2012 to more than 70 years in 2040.

Our study provides insights on the trends in HCC in the DAA era. Some studies^39,40,41^ conducted statistical analysis based on cancer registry data predominately in the pre-DAA era. The forecasted incidence was based on historical trends without factoring in the changes in the management of HCV.^39,42^ Thus those findings may not be applicable to the DAA era. While other studies that used DAA-era data reported that the burden of HCC will decrease in the era of DAAs,^8,13,43^ those studies excluded patients with HCV who were incarcerated, homeless, and residing in nursing homes, and were active-duty military personnel. Our study accounted for such populations with less access to screening and treatment for HCV than the general population, and we found that the overall HCC incidence in the population with HCV will continue to increase until 2021 despite the recent availability of highly effective DAAs.

Our study also provides information on the shifting burden of surveillance for HCC and the need to emphasize the clinical treatment for patients with virologically cured HCV. Despite virological cure, patients with advanced fibrosis or cirrhosis will remain at risk of developing HCC. Therefore, as the number of patients with virologically cured HCV increases, the new cases of HCC from this cohort will increase. In addition, the burden of surveillance for HCC will shift from patients with viremia to patients with virologically cured HCV. These changes may have substantial practical implications for the continuum of care. Most of the patients with virologically cured HCV will transition from receiving care from liver specialists to receiving care from primary care physicians. However, most people do not receive regular surveillance in primary care settings^44^ and the knowledge of HCC surveillance in primary care settings remains low.^45^ Therefore, it is vital to emphasize that biannual HCC surveillance is warranted for these patients in primary care settings.

The optimal clinical management of patients with virologically cured HCV remains unclear. While the HCC management guidelines by the European Association for the Study of the Liver recommends routine HCC surveillance in patients with virologically cured HCV having advanced fibrosis and cirrhosis,^7^ the guideline by the American Association for the Study of Liver Diseases recommends routine HCC surveillance only in individuals with cirrhosis without specifying the status of virological cure.^6^

The optimal surveillance strategy in patients with virologically cured HCV will rely on more evidence and studies in the following 3 aspects. First, long-term data are needed for a better understanding of the risk of HCC after DAA-induced virological cure in patients with HCV. It is unclear how much of the current knowledge of the HCC risk among patients with virologically cured HCV from an interferon-based regimen could be used to inform surveillance policies in the era of DAAs. Some studies suggest that patients with HCV cured by DAA regimens could have higher HCC risks than those with HCV cured by interferon-based regimens,^9,10,46^ but the increase was not found to be significant after adjusting for confounders,^47^ and the difference was not found to be conclusive.^48^ In addition, compared with the available data in the interferon-based treatment era, the observations in DAA era are subject to shorter follow-up time and present an older at-risk population.

Second, health economic data are needed to identify cost-effective HCC surveillance policies for patients with virologically cured HCV. The primary reason for not recommending routine surveillance in patients with virologically cured HCV with advanced fibrosis may be that biannual surveillance using ultrasonography and α_1_-fetoprotein testing for the lifetime was not found to be cost-effective in this cohort.^49,50^ Instead of a general surveillance, further research is warranted to identify risk-based surveillance in these patients that could provide a good use of limited resources.^50^ In addition, the optimal age for stopping surveillance is unknown. It is plausible that patients with virologically cured HCV may not need HCC surveillance throughout their remaining lifetime.

Third, tailoring surveillance policies to individual-level factors may be a useful approach, especially as emerging molecular biomarkers show their value in the prediction of HCC risks in addition to current clinical risk factors.^51^ Comprehensive risk prediction models can be developed by integrating multilevel risk factors to further refine the risk predictions.^52^ A tailored surveillance strategy could differentiate the patients at high risk who can benefit more from surveillance compared with the others, and thus further improve the cost-effectiveness and efficiency of medical resource use.^53,54^

Like any modeling study, our model projection results are subject to inherent uncertainty that arises from the uncertainties in model parameter estimates and model assumptions. Some model parameters and assumptions cannot capture all possible disease variability and diverse real-world clinical practice. Therefore, instead of presenting the prediction of outcomes, our model projection results provide insights on the overall trends of the HCC incidence and surveillance burden, which has also been shown to be robust in the presence of model uncertainty from our sensitivity analysis.

### Limitations

This study has limitations. First, we did not explicitly model HCC diagnosis process, which depends on incidence, underlying surveillance strategies, and testing accuracy. We considered HCC as an aggregate health state and used aggregate-level parameters for the population to characterize the overall burden of HCC. Further details would be necessary for analyzing the outcomes of particular HCC surveillance and treatment strategies. Second, our analysis was limited to HCV-associated HCC and did not account for other emerging risk factors such as metabolic disorder (eg, diabetes, nonalcoholic fatty liver disease), hepatitis B, and alcohol consumption. Management of HCC in these other populations at increased risk is becoming increasingly important but would require modeling of different disease factors, which is beyond the scope of this study. In addition, our model did not account for fibrosis regression following successful DAA treatment. Based on recent studies that suggested stable HCC risks following SVR in patients including those with improved noninvasive markers for fibrosis,^55,56^ we assumed that patients would still be candidates for HCC surveillance even if they had fibrosis regression. However, it is possible that the risk of HCC could decrease with fibrosis regression as time from SVR accrues; therefore, our study may have overestimated the burden of HCC surveillance.

## Conclusions

This decision analytical model study suggests that the new cases of HCC associated with HCV will continue to increase even in the era of DAAs. We also found that the risk of HCC as well as the burden of HCC surveillance is projected to shift from patients with active HCV to those who had achieved virological cure. Routine HCC surveillance in patients with virologically cured HCV who remain at risk of HCC may help reduce the burden of HCC in the era of new antiviral agents.

## Notes

Supplement. **eTable 1.** Population Characteristics of Hepatitis C Patients in the United States **eTable 2.** Age Distribution of Initial Population Infected With Hepatitis C **eTable 3.** Annual Hepatitis C Incidence for Years 2001 to 2040 **eTable 4.** State Transition Probabilities Used in HEP-SIM **eTable 5.** Screening Type and Annual Rate by Subpopulation **eTable 6.** Market Share of NS5A Inhibitors (Versus Non-NS5A Inhibitors) Inhibitors by Year of Treatment **eTable 7.** SVR Rates by Treatment, Genotype, Treatment History, and Fibrosis States **eTable 8.** Treatment Uptake Rate by Subpopulation **eTable 9.** Insurance Status of Hepatitis C Patients Under Age 65 **eTable 10.** Insurance Changes According to the Affordable Care Act **eTable 11.** Sampling Distribution for Model Parameters in Probabilistic Sensitivity Analyses **eFigure 1.** Annual HCC Incidence by Cirrhosis Status and SVR Status **eFigure 2.** Probabilistic Sensitivity Analysis Results for the Number of HCC Incidence and Surveillance Candidates by Cure Status **eFigure 3.** Number of Candidates for HCC Surveillance by Fibrosis Stage, 2012-2040 **eFigure 4.** The Average Age of HCC Incidence During 2012-2040 in DAA Era **eReferences**
