# Supplementary material for: Assessment of Incidence of and Surveillance Burden for Hepatocellular Carcinoma Among Patients With Hepatitis C in the Era of Direct-Acting Antiviral Agents
Source: JAMA Netw Open. 2020 Nov 18;3(11):e2021173. doi: 10.1001/jamanetworkopen.2020.21173 (PMC7675109; doi:10.1001/jamanetworkopen.2020.21173)
Supplement: Supplement. — eTable 1. Population Characteristics of Hepatitis C Patients in the United States eTable 2. Age Distribution of Initial Population Infected With Hepatitis C eTable 3. Annual Hepatitis C Incidence for Years 2001 to 2040 eTable 4. State Transition Probabilities Used in HEP-SIM eTable 5. Screening Type and Annual Rate by Subpopulation eTable 6. Market Share of NS5A Inhibitors (Versus Non-NS5A Inhibitors) Inhibitors by Year of Treatment eTable 7. SVR Rates by Treatment, Genotype, Treatment History, and Fibrosis States eTable 8. Treatment Uptake Rate by Subpopulation eTable 9. Insurance Status of Hepatitis C Patients Under Age 65 eTable 10. Insurance Changes According to the Affordable Care Act eTable 11. Sampling Distribution for Model Parameters in Probabilistic Sensitivity Analyses eFigure 1. Annual HCC Incidence by Cirrhosis Status and SVR Status eFigure 2. Probabilistic Sensitivity Analysis Results for the Number of HCC Incidence and Surveillance Candidates by Cure Status eFigure 3. Number of Candidates for HCC Surveillance by Fibrosis Stage, 2012-2040 eFigure 4. The Average Age of HCC Incidence During 2012-2040 in DAA Era eReferences [file jamanetwopen-e2021173-s001.pdf]

Supplemental Online Content

Chen Q, Ayer T, Adee MG,Wang X, Kanwal F, Chhatwal J. Assessment of incidence of and surveillance burden for hepatocellular carcinoma among patients with hepatitis C in the era of direct-acting antiviral agents. *JAMA Netw Open*. 2020;3(11):e2021173. doi:10.1001/jamanetworkopen.2020.21173

eTable 1. Population Characteristics of Hepatitis C Patients in the United States

eTable 2. Age Distribution of Initial Population Infected With Hepatitis C

eTable 3. Annual Hepatitis C Incidence for Years 2001 to 2040

eTable 4. State Transition Probabilities Used in HEP-SIM

eTable 5. Screening Type and Annual Rate by Subpopulation

eTable 6. Market Share of NS5A Inhibitors (Versus Non-NS5A Inhibitors) Inhibitors by Year of Treatment

eTable 7. SVR Rates by Treatment, Genotype, Treatment History, and Fibrosis States

eTable 8. Treatment Uptake Rate by Subpopulation

eTable 9. Insurance Status of Hepatitis C Patients Under Age 65

eTable 10. Insurance Changes According to the Affordable Care Act

eTable 11. Sampling Distribution for Model Parameters in Probabilistic Sensitivity Analyses

eFigure 1. Annual HCC Incidence by Cirrhosis Status and SVR Status

eFigure 2. Probabilistic Sensitivity Analysis Results for the Number of HCC Incidence and Surveillance Candidates by Cure Status

eFigure 3. Number of Candidates for HCC Surveillance by Fibrosis Stage, 2012-2040

eFigure 4. The Average Age of HCC Incidence During 2012-2040 in DAA Era

eReferences

This supplemental material has been provided by the authors to give readers additional information about their work.

© 2020 Chen Q et al. *JAMA Network Open*.

Supplement S1: Model Details and Inputs

HEP-SIM Model Details

We developed an individual-level state-transition model, Hepatitis C Disease Burden Simulation model (HEP-SIM) that simulated chronic hepatitis C virus (HCV)-infected population in the United States from 2001 to 2040 (1, 2). The model simulated the clinical course of patients with chronic HCV infection, including new incidence of infection, natural history of disease progression, diagnosis and treatment. HEP-SIM was implemented in C++ programming language for computation efficiency. More model details can be found elsewhere (2).

We defined the stage of HCV infection using the METVIR scoring system (no fibrosis [F0], portal fibrosis without septa [F1], portal fibrosis with few septa [F2], numerous septa without cirrhosis [F3], or cirrhosis [F4]). Patients could develop the adverse outcomes of decompensated cirrhosis (DC) and/or hepatocellular carcinoma (HCC), could receive a liver transplant (LT), or experience a liver-related death (LRD). Patients who achieved sustained virologic response (SVR) were assumed to transition into normal health status only if their METAVIR stage is lower than or equal to stage F3. For cirrhotic (METAVIR stage F4) patients, we assumed that the disease would continue progressing even after achieving SVR, though at a slower rate (3).

In this appendix, we summarize the key model parameters and assumptions. Table S1 provides the characteristics of HCV-infected patients, including both NHANES and non-NHANES population, that are simulated in HEP-SIM. Table S3 summarizes age distribution of HCV population, and Table S3 summarizes input parameters for annual incidences of HCV infection. Table Sr includes the transition probability parameters for natural history of HCV.

eTable 1. Population characteristics of hepatitis C patients in the United States

Parameter	Value	Reference
Total HCV seropositive number		
NHANES population in 2015	2,127,600	(4)
Incarcerated in 2006 [a]	1.45 million	(5)
Homeless in 2015	17,400	(4)
Nursing home in 2015	6,900	(4)
Active-duty military in 2015	6,900	(4)
Male (%)		
NHANES	64.2%	(4)
Incarcerated	87.8%	(6)
Homeless [b]	74.5%	(6)
Hospitalized/nursing home	64.2%	(4)
Active military	90.9%	(4)
Fibrosis distribution at 2015 [c]		www.MappingHepC.com
F0-F1 stage	44.2%	
F2 stage	28.5%	
F3 stage	11.2%	
Compensated cirrhosis (F4)	16.1%	
Genotype distribution		(7)
Genotype 1	73%	
Genotype 2	14%	
Genotype 3	8%	
Genotypes 4-6	5%	
Standardized mortality ratio (adjustment for background mortality) for homeless	4.4	(8)
Baseline awareness rate for age below 65[d]		
Incarcerated population (year 2015)	25%	(9, 10)

© 2020 Chen Q et al. *JAMA Network Open.*

NHANES population (year 2015)		(11)
Insured, age <40	17.6%	
Insured, age 40-49	60.5%	
Insured, age 50-59	54.6%	
Insured, age ≥60	34.9%	
Uninsured, age <40	5.8%	
Uninsured, age 40-49	19.5%	
Uninsured, age 50-59	16.3%	
Uninsured, age ≥60	5.77%	
Nursing home, military, homeless population (added at 2015)		Calibrated to the awareness rate in 2015
New HCV incidences	7.2%	(12)

[a] We assumed that 78% of 1,857,000 of HCV antibody persons in corrections were chronic
[b] Assumed same as in NHANES population
[c] We assumed the same fibrosis distribution across non-NHANES population
[d] HCV patients above age 65 were assumed to have insured from Medicare

eTable 2. Age distribution of initial population infected with hepatitis C

NHANES in 2001[a] (11, 13)		Homeless population (14)		Nursing home (15)		Military population (16)		Incarcerated (9)		New incidence (17)	
**Age group**	**%**	**Age group**	**%**	**Age group**	**%**	**Age group**	**%**	**Age group**	**%**	**Age group**	**%**
**18-19**	1.8%	18-29	5.0%	50-64	15.0%	18-25	50.8%	18	0.1%	18-19	3.2%
**20-29**	10.7%	30-44	36.6%	65-74	14.6%	26-30	21.3%	20	1.9%	20-29	34.9%
**30-39**	22.7%	45-59	55.9%	75-84	27.5%	31-35	14.0%	30	17.5%	30-39	32.9%
**40-49**	28.9%	60+	2.5%	85-96	35.3%	36-40	8.8%	40	51.3%	40-49	18.2%
**50-59**	20.4%			95+	7.6%	41+	5.1%	50	27.6%	50-59	7.1%
**60-69**	9.3%							60+	1.6%	60+	3.6%
**70+**	6.2%										

eTable 3. Annual hepatitis C incidence for years 2001 to 2040

Year	Annual HCV Incidence[a]
2001	24,000
2002	29,000
2003	28,000
2004	26,000
2005	21,000
2006	19,000
2007	17,000
2008	18,000
2009	16,000
2010	17,000
2011	16,500
2012	24,700
2013	29,700
2014	30,500
2015	33,900
**Projected HCV incidence**	
2016	41,200
2017	43,420
2018	45,640
2019	47,860
2020	50,080

© 2020 Chen Q et al. *JAMA Network Open.*

2021	52,300
2022	54,520
2023	56,740
2024	58,960
2025	61,180
2026	63,400
2027-2040	63,400

[a]New incidence are distributed among NHANES and prison population, based on the prevalence in the previous year

eTable 4. State transition probabilities used in HEP-SIM

Transition probabilities	Value	Reference
F0 to F1	0.117	(18)
F1 to F2	0.085	(18)
F2 to F3	0.120	(18)
F3 to F4	0.116	(18)
F3 to HCC	0.0063	(19)
F3-SVR to HCC	0.0034	(20)
F4 to DC	0.039	(21)
F4 to HCC	0.021	(19)
F4-SVR to DC	0.008	(3)
F4-SVR to HCC	0.0182	(20)
DC to HCC	0.068	(22)
DC-SVR to HCC	0.0361	(23)
DC to LT	0.023	(24, 25)
DC (first year) to death from liver disease	0.182	(22)
DC (subsequent years) to death from liver disease	0.112	(22)
HCC to LT	0.040	(26, 27)
HCC to death from liver disease	0.427	(21)
LT (first year) to death from liver disease	0.116	(28)
PLT to death from liver disease	0.044	(28)

F0 = no fibrosis; F1 = portal fibrosis without septa; F2 = portal fibrosis with few septa; F3 = numerous septa without cirrhosis; F4 = cirrhosis; DC = decompensated cirrhosis; HCC = hepatocellular carcinoma; LT = liver transplantation (first year); PLT = post liver transplantation (> 1 year).

HCV Screening

Unaware individuals could become aware of their HCV infection by usual care (risk-based diagnostic testing), birth cohort screening, or universal screening. We assumed the screening rate by usual care in nursing home, military, and homeless population identical to that of NHANES population. From 2013 onwards, the birth-cohort screening included one-time screening of baby boomers (i.e., those born between 1945 and 1965) among NHANES, nursing home, and homeless population. One-time universal HCV screening in all adults (NHANES and non-NHANES population) replaces birth-cohort screening from 2020 onwards.

eTable 5. Screening type and annual rate by subpopulation

Screening type	NHANES	Nursing home	Military	Homeless	Incarcerated
**Usual care**	2%/year	2%/year	2%/year	2%/year	1% up to year 2015,<br>2.5% from 2016 onwards
**Birth cohort[1] (29)**	3%/year for 2013-2015, 9% from 2016 onwards	3%/year for 2013-2015, 9% from 2016 onwards	3%/year for 2013-2015, 9% from 2016 onwards	--	--

© 2020 Chen Q et al. *JAMA Network Open*.

**Universal[1] (29)**	9%/year from 2020 onwards	9%/year from 2020 onwards	9%/year from 2020 onwards	9%/year from 2020 onwards	9%/year from 2020 onwards

[1]Implemented in addition to usual care screening

HCV Treatment Waves

We modeled HCV treatment in different waves reflecting clinical practice starting with peginterferon+ribavirin until 2011, followed by the launch of first-generation protease inhibitors, boceprevir and telaprevir, in 2011. From 2014, we simulated the availability of non-NS5A inhibitors sofosbuvir and simeprevir (denoted as DAA1 non-NS5A wave). From 2015, we simulated all-oral DAA combinations including both non-NS5A and NS5A inhibitors (denoted by DAA1 non-NS5A and DAA1 NS5A), followed by the availability of next wave of NS5A inhibitors (denoted by DAA2 NS5A) from 2018 onwards. Market share of different types of treatment regimens by HCV genotype was estimated from commercial claims from QuintilesIMS and IPSOS (Table S6). We obtained SVR rates from multiple clinical trials as well as real-world data from the TRIO and TARGET studies (Table S7).

eTable 6. Market share of NS5A inhibitors (versus non-NS5A inhibitors) inhibitors by year of treatment

	Genotype 1	Genotype 2	Genotype 3	Genotype 4–6
**Before 2014**	0%	0%	0%	0%
**2015**	90%	10%	30%	61%
**2016**	90%	10%	30%	61%
**After 2017**	100%	90%	90%	100%

Note: the market share is based on the year the patients finished treatment with a given therapy and not based on the time the drugs were approved.

eTable 7. SVR rates by treatment, genotype, treatment history, and fibrosis states

Treatment history and fibrosis state	GT1	GT2	GT3	GT4–6	References
**PEG+RBV**					
Treatment naïve					(1, 30-34)
F0-F3	0.54	0.82	0.70	0.58	
F4	0.36	0.64	0.49	0.32	
Contraindicated with modifiable reasons					
F0-F2	-	0.66	0.56	0.46	
F3	0.43	0.66	0.56	0.46	
F4	0.28	0.51	0.40	0.26	
Failed PEG+RBV: relapse					
F0-F3	0.27	0.71	0.66	0.31	
F4	0.13	0.56	0.52	0.24	

© 2020 Chen Q et al. *JAMA Network Open.*

Failed PEG+RBV: partial response					
F0-F3	0.18	0.69	0.64	0.31	
F4	0.10	0.55	0.51	0.24	
Failed PEG+RBV: null response					
F0-F3	0.10	0.54	0.50	0.31	
F4	0.05	0.42	0.39	0.24	
**BOC/TEL+PEG+RBV (GT1 only)**					
Treatment naïve					(1, 35-38)
F0-F3	0.75	-	-	-	
F4	0.62	-	-	-	
Contraindicated with modifiable reasons					
F0-F2	-	-	-	-	
F3	0.5	-	-	-	
F4	0.36	-	-	-	
Failed PEG+RBV: relapse					
F0-F2	0.87	-	-	-	
F3	0.85	-	-	-	
F4	0.84	-	-	-	
Failed PEG+RBV: partial response					
F0-F2	0.72	-	-	-	
F3	0.56	-	-	-	
F4	0.56	-	-	-	
Failed PEG+RBV: null response					
F0-F2	0.41	-	-	-	
F3	0.39	-	-	-	
F4	0.14	-	-	-	
**DAA non-NS5A[1]**					(39-42)
Treatment naïve					
F0-F3	0.9	0.9	0.85	0.9	
F4	0.8	0.8	0.6	0.8	
Contraindicated with modifiable and non-modifiable reasons					
F0-F3	0.9	0.9	0.9	0.9	
F4	0.7	0.7	0.6	0.7	
Failed PEG+RBV: relapse					
F0-F3	0.9	0.9	0.85	0.9	
F4	0.8	0.7	0.6	0.75	
Failed PEG+RBV: partial and null response					
F0-F3	0.9	0.9	0.85	0.9	
F4	0.75	0.7	0.6	0.75	
Failed first-generation PI					
F0-F3	0.9	-	-	-	
F4	0.7	-	-	-	
Failed DAA NS5A (during 2015-2018)					

© 2020 Chen Q et al. *JAMA Network Open*.

F0-F3	-	-	-	-	
F4	0.8	0.8	0.6	0.8	
**DAA NS5A[2]**					
Treatment naïve, contraindicated, failed PEG+RBV, failed BOC/TEL+PEG+RBV (GT1 only), failed DAA non-NS5A, failed DAA nonNS5A					(43-51)
F0-F3	0.95	0.99	0.95	0.99	
F4	0.9	0.99	0.9	0.99	
**DAA NS5A-next generation[3]**					
All conditions					(52-56)
F0-F3	0.95	0.99	0.95	0.99	
F4	0.9	0.99	0.9	0.99	

[1]DAA1 non-NS5A includes the following drug combinations: SOF+IFN+/-RBV, SOF+/-RBV, SOF+SMV+/-RBV, and SMV+IFN+/-RBV.
[2]DAA1 NS5A includes the following drug combinations: SOF+LDV+/-RBV, SOF+DCV, DCV+IFN+/-RBV, OBV/PTV/r+DSV+/-RBV, OBV/PTV/r+/-RBV, EBR+GZR, and SOF+VEL.
[3]DAA2 NS5A includes the next wave of potential drug combinations such as SOF/VEL/VOX, grazoprevir/ruzasvir/uprifosbuvir, glecaprevir/pibrentasivr, and odalasvir+AL-335+/SMV

HCV treatment uptake rate

If patients were aware of their disease and had access to insurance, they were considered candidates for receiving treatments. We used a previously published study to estimate the number of patients who received treatment between 2001 and 2007 (57) among NHANES population, active duty military and nursing home population, and then we assumed that the treatment rates remained steady between 2008 and 2013. In 2014, 141,000 persons in the United States received HCV treatment with sofosbuvir-based therapies (58). Assuming 90% market share of sofosbuvir and ledipasvir, we found that the total number of patients who started treatment in 2014 to be around 150,000. In 2015, approximately 280,000 initiated treatment based on the drug sales (59). We assumed that the maximum treatment uptake rate would remain at 280,000/year beyond 2015. In incarcerated and homeless populations, treatment uptake was generally low, which was assumed to be 1% per year up to 2016 and 5% per year since 2017 (based on data from www.HepCorrections.org).

Patients with decompensated cirrhosis were not eligible for treatment. Prior to 2018, patients who had failed NS5A DAA could be re-treated only if they were cirrhotic; otherwise they had to wait until 2018 when the next generation NS5A DAA (DAA2 NS5A) became available. From 2018 onwards, all patients were eligible for re-treatment with DAA2 NS5A inhibitors after failing previous DAAs

eTable 8 Treatment uptake rate by subpopulation

Parameter	Annual Treatment Uptake	Reference
NHANES, nursing homes, and active-duty military		
2001	126,040	(57)
2002	126,040	(57)
2003	107,131	(57)
2004	144,276	(57)
2005	114,197	(57)
2006	88,083	(57)
2007	83,270	(57)

2008-2013	83,270	Assumption
2014	150,000	(58)
2015	280,000	(59)
2016-2040	280,000	Assumption
Incarcerated population and homeless		
2010-2016	1%	(60)
From 2017 and onwards	5%	Assumption

eTable 9. Insurance status of hepatitis C patients under age 65

Sub population	Value	Reference
NHANES population		(11, 61, 62)
Private insurance	49.8%	
Medicaid	14.3%	
Other public insurance	9.2%	
Uninsured	26.7%	
Homeless population		(63)
Private insurance	3.2%	
Medicaid	31.5%	
Uninsured	65.3%	
Nursing home	Same as NHANES population	Assumption
Prisons		
Separate insurance type	100%	Assumption
Active-duty military		
Other public insurance	100%	Assumption

eTable 10. Insurance changes according to the Affordable Care Act

	2014	2015	2016	2017
Cumulative percentage of uninsured who got insurance under ACA (compared to 2013)	22.2%	35.2%	46.3%	48.1%
Percentage of newly added to Private Exchanges (compared to preceding year)	+10.3%	+7.0%	+7.4%	+1.3%
Percentage of newly added to Medicaid (compared to preceding year)	+12.0%	+5.9%	+3.7%	+0.6%

We incorporated changes in the insurance pool because of the implementation of the Affordable Care Act (ACA) using a report by the Congressional Budget Office and the staff of the Joint Committee on Taxation (64). Table shows changes in uninsured population after the implementation of ACA in 2014 and beyond. The table also shows patients who got insurance through Medicaid

Probabilistic Sensitivity Analysis

We performed probabilistic sensitivity analyses (PSA) to assess impact of input parameter uncertainty on the model results. Sampling distributions for model parameter uncertainty are defined in **Table S11**.

For model parameters that represent a probability (such as state transition probability, awareness rate, percentage of male, diagnosis rates, and the chronic infection ratio), we assumed Beta distribution for sampling the parameter values in the PSA. The mean μ of the Beta distribution was determined by the baseline value, and the standard deviation σ is determined by the 1/4 range of the reported confidence interval if available (assuming the confidence interval covers the range $\mu \pm 2\sigma$), or 20% of baseline value μ. For several SVR estimates with very high baseline values (e.g., 0.95, and 0.99, mostly for NS5A DAAs), the Beta distribution with $\sigma = 20\%\mu$ is not properly defined. Instead, we simply assumed that the SVR

© 2020 Chen Q et al. *JAMA Network Open.*

values that have a baseline value >0.9 vary uniformly within ±0.05 range (truncated by maximum value of 1.0), and other values follow the Beta distribution with σ/μ=10%.

For other parameters that represent a probability distribution (rather than a point estimate), such as age, fibrosis, genotype distributions, we assumed these parameters follow Dirichlet distribution. In particular, given a distribution estimate $(p_1, p_2, \cdots, p_N) \sim \mathrm{Dir}(\alpha_1, \cdots, \alpha_N)$, the variance of each component can be calculated as $Var[p_k] = \frac{\alpha_k(\alpha_0 - \alpha_k)}{\alpha_0^2(\alpha_0+1)}$. We estimated the parameters $(\alpha_1, \cdots, \alpha_N)$ by setting the ratio of standard deviation to mean (σ/μ) to be at most 20%.

We performed PSA with 5000 samples. Model output uncertainty was represented by 95% uncertainty interval defined as 2.5th -97.5th percentile for each model outcome.

eTable 11. Sampling distribution for model parameters in probabilistic sensitivity analyses

Parameter	Range	Distribution	Parameter 1	Parameter 2
Probability estimates				
F0 to F1	0.104 - 0.13	Beta	285.98	2158.26
F1 to F2	0.075 - 0.096	Beta	239.77	2581.00
F2 to F3	0.109 - 0.133	Beta	351.88	2580.45
F3 to F4	0.104 - 0.129	Beta	304.40	2319.73
F4 to DC	0.01 - 0.079	Beta	4.87	120.08
Post-SVR F4 to DC	0.002 - 0.036	Beta	0.87	107.97
F3 to HCC	0.005 - 0.008	Beta	70.11	11058.33
Post-SVR F3 to HCC	0.002 - 0.005	Beta	20.48	6002.42
F4 to HCC	0.014 - 0.03	Beta	26.96	1256.97
Post-SVR F4 to HCC	0.015 - 0.021	Beta	144.52	7796.15
DC to HCC	0.03 - 0.083	Beta	24.48	335.51
DC to liver transplant	0.01 - 0.062	Beta	3.04	128.93
Mortality of DC in first year	0.065 - 0.19	Beta	27.56	123.89
Mortality of DC in subsequent years	0.065 - 0.19	Beta	11.29	89.55
HCC to liver transplant	0 - 0.14	Beta	1.21	29.13
Mortality of HCC	0.33 - 0.86	Beta	5.52	7.41
Mortality of liver transplant in first year	0.06 - 0.42	Beta	1.35	10.31
Mortality of liver transplant in subsequent years	0.024 - 0.11	Beta	3.96	86.04
Chronic infection ratio	0.704 - 0.866	Beta	80.82	22.80
Proportion of DC patients eligible for transplant	0.05-0.25	Beta	7.5	42.5
Awareness ratio among new incidences		Beta[a]	0.20	-
Awareness ratio in the initial population		Beta[a]	0.20	-
Male %		Beta[a]	0.20	-
Diagnosis rate (usual care)		Beta[a]	0.20	-
SVR for other treatment regimens		Beta[a]	0.10	-
SVR for NS5A DAAs (changes to the baseline value)		Uniform	-0.05	+0.05
Distribution estimates				
Genotype		Dirichlet[a]	0.20	-

© 2020 Chen Q et al. *JAMA Network Open.*

Age of initial population	Dirichlet[a]	0.20	-
Age of new HCV incidences	Dirichlet[a]	0.20	-
Fibrosis state of initial population	Dirichlet[a]	0.20	-

[a] Distribution parameters depend on parameter baseline values. Parameters for the Beta distribution were estimated by assuming the standard deviation-mean ratio (σ/μ) = 20%. Parameters for the Dirichlet distribution were estimated by assuming the largest standard deviation-mean ratio ($\max_k \sigma_k/\mu_k$) = 20%.

© 2020 Chen Q et al. *JAMA Network Open*.

Supplement S2. Additional Model Results

eFigure 1. Annual HCC incidence by cirrhosis status and SVR status.

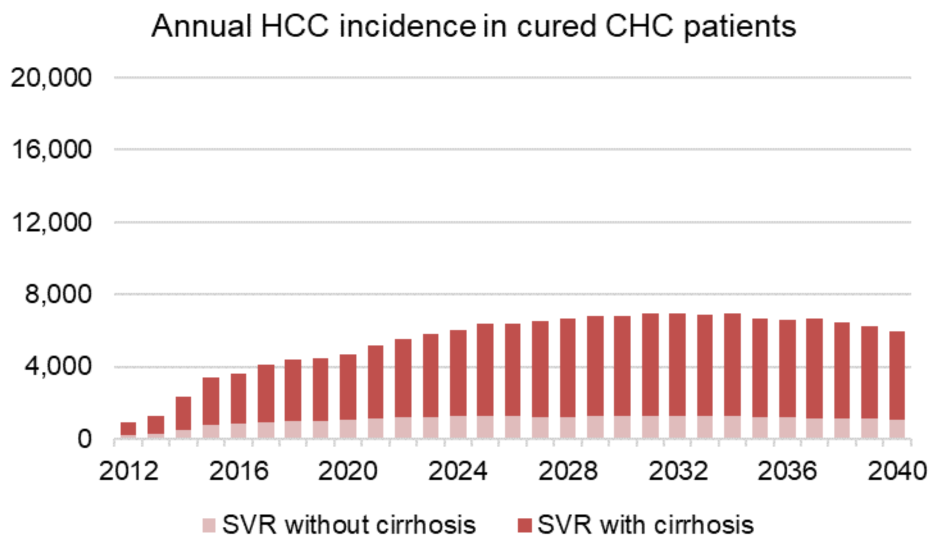

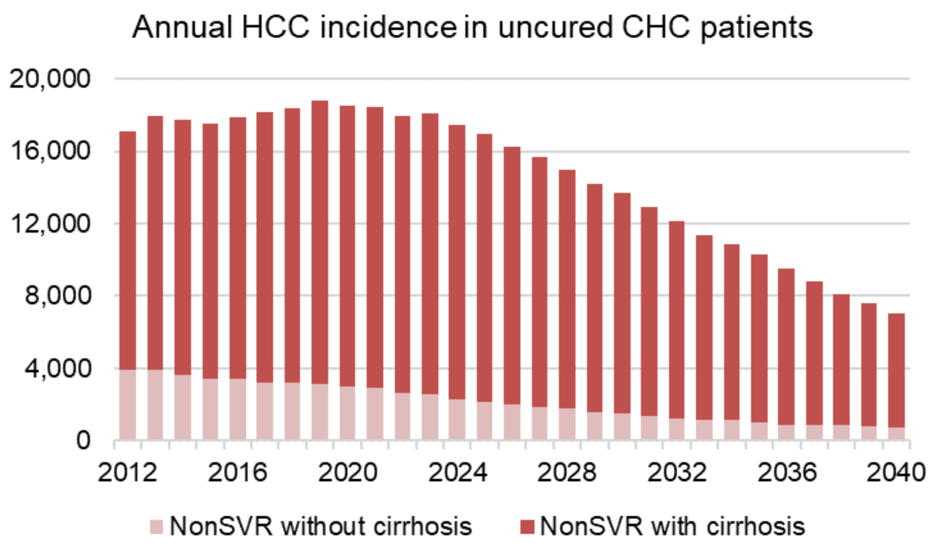

© 2020 Chen Q et al. *JAMA Network Open*.

eFigure 2. Probabilistic sensitivity analysis results for the number of HCC incidence and surveillance candidates by cure status.

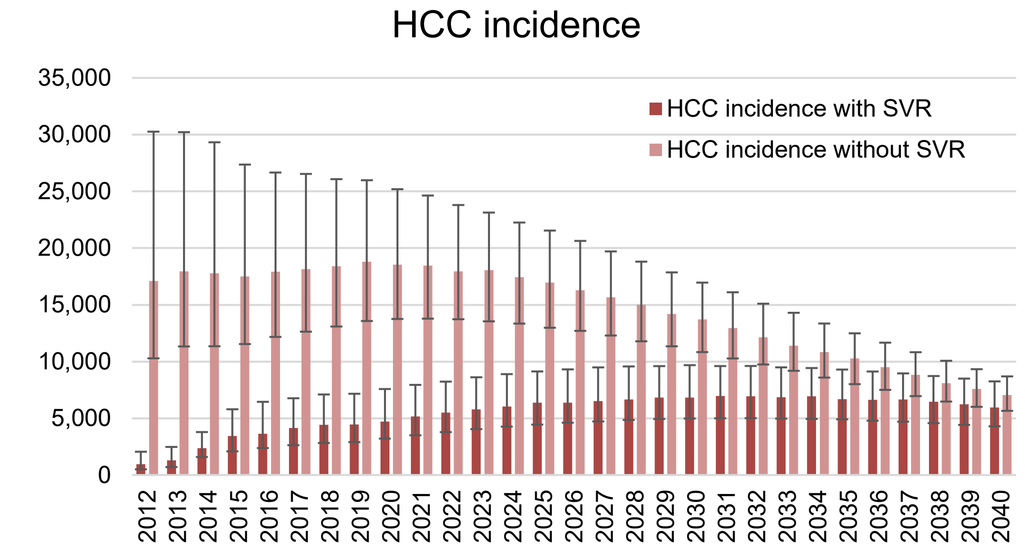

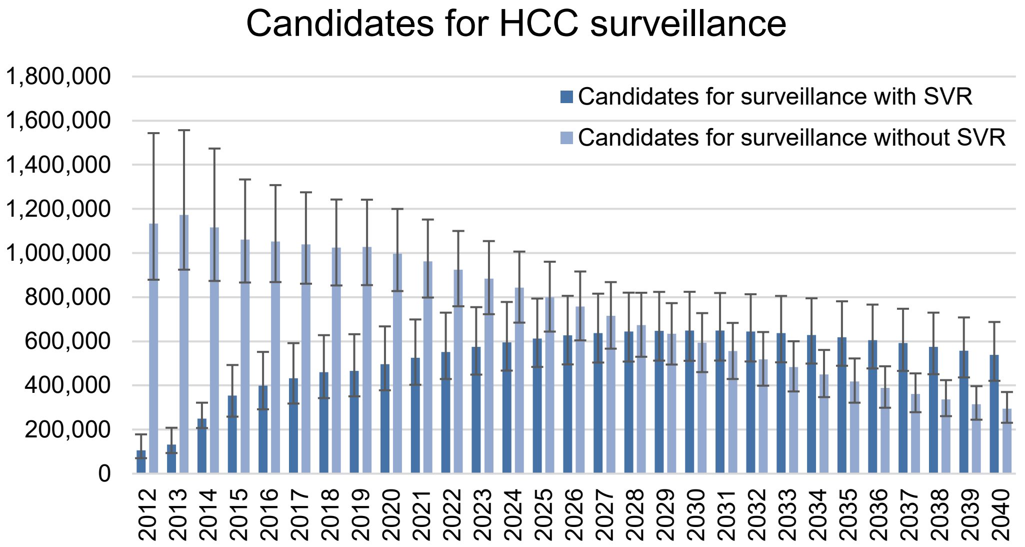

© 2020 Chen Q et al. *JAMA Network Open*.

eFigure 3. Number of candidates for HCC surveillance by fibrosis stage, 2012-2040.

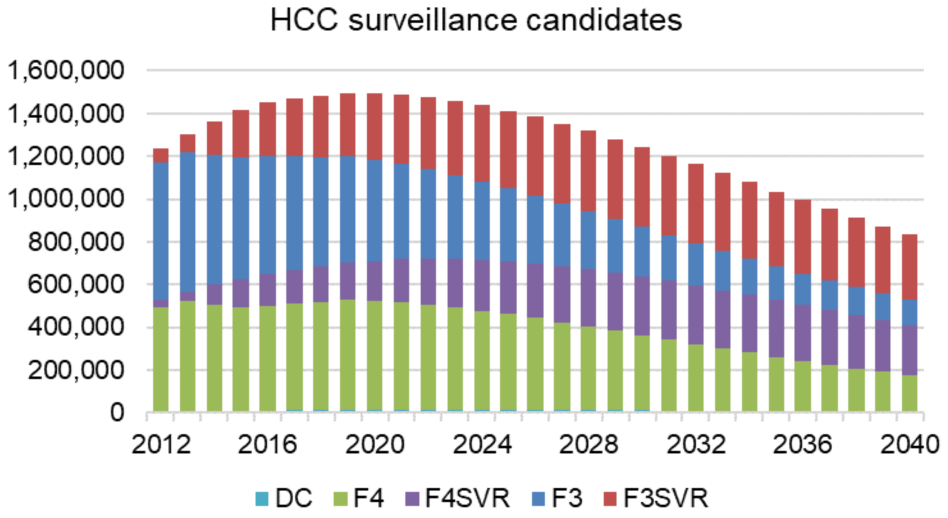

eFigure 4. The average age of HCC incidence during 2012-2040 in DAA era.

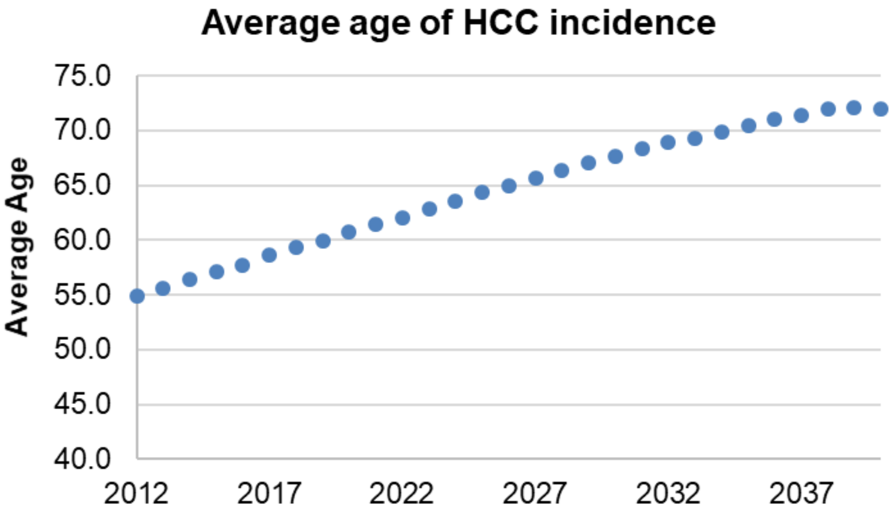

© 2020 Chen Q et al. *JAMA Network Open*.

eReferences

1. Kabiri M, Jazwinski AB, Roberts MS, Schaefer AJ, Chhatwal J. The changing burden of hepatitis C in the United States: Model-based predictions. Annals of Internal Medicine. 2014;161(3):170-80.
2. Chhatwal J, Chen Q, Bethea ED, Hur C, Spaulding AC, Kanwal F. The Impact of Direct-Acting Antivirals on the Hepatitis C Care Cascade: Identifying Progress and Gaps towards Hepatitis C Elimination in the United States. Alimentary Pharmacology & Therapeutics. 2019;In press.
3. Cardoso AC, Moucari R, Figueiredo-Mendes C, Ripault MP, Giuily N, Castelnau C, et al. Impact of peginterferon and ribavirin therapy on hepatocellular carcinoma: incidence and survival in hepatitis C patients with advanced fibrosis. Journal of hepatology. 2010;52(5):652-7.
4. Hofmeister MG, Rosenthal EM, Barker LK, Rosenberg ES, Barranco MA, Hall EW, et al. Estimating Prevalence of Hepatitis C Virus Infection in the United States, 2013-2016. Hepatology. 2018;Epub ahead of print.
5. Varan AK, Mercer DW, Stein MS, Spaulding AC. Hepatitis C seroprevalence among prison inmates since 2001: still high but declining. Public Health Reports. 2014;129(2):187-95.
6. Edlin BR, Eckhardt BJ, Shu MA, Holmberg SD, Swan T. Toward a more accurate estimate of the prevalence of hepatitis C in the United States. Hepatology. 2015;62(5):1353-63.
7. Blatt LM, Mutchnick MG, Tong MJ, Klion FM, Lebovics E, Freilich B, et al. Assessment of hepatitis C virus RNA and genotype from 6807 patients with chronic hepatitis C in the United States. J Viral Hepat. 2000;7(3):196-202.
8. Morrison DS. Homelessness as an independent risk factor for mortality: results from a retrospective cohort study. International Journal of Epidemiology. 2009;38(3):877-83.
9. He T, Li K, Roberts MS, Spaulding AC, Ayer T, Grefenstette JJ, et al. Prevention of Hepatitis C by Screening and Treatment in US PrisonsPrevention of Hepatitis C in US Prisons. Annals of internal medicine. 2016;164(2):84-92.
10. Spaulding AC, Thomas DL. Screening for HCV infection in jails. Jama. 2012;307(12):1259-60.
11. Chhatwal J, Wang X, Ayer T, Kabiri M, Chung RT, Hur C, et al. Hepatitis C disease burden in the United States in the era of oral direct‐acting antivirals. Hepatology. 2016;64(5):1442-50.
12. CDC. Viral Hepatitis: Statistics and Surveillance 2016 [Available from: https://www.cdc.gov/hepatitis/hcv/statisticshcv.htm.
13. Armstrong GL, Wasley A, Simard EP, McQuillan GM, Kuhnert WL, Alter MJ. The prevalence of hepatitis C virus infection in the United States, 1999 through 2002. Annals of internal medicine. 2006;144(10):705-14.
14. Strehlow AJ, Robertson MJ, Zerger S, Rongey C, Arangua L. Hepatitis C among clients of health care for the homeless primary care clinics. Journal of health care for the poor and underserved. 2012;23(2):811.
15. Centers for Medicare & Medicaid Services. Nursing Home Data Compendium 2015 Edition. 2015.
16. US Department of Defense. 2016 Demographics: Profile of the military community. 2016 [Available from: http://download.militaryonesource.mil/12038/MOS/Reports/2016-Demographics-Report.pdf.
17. CDC. Surveillance for Viral Hepatitis – United States, 2014 2016 [Available from: https://www.cdc.gov/hepatitis/statistics/2014surveillance/index.htm#tabs-1170600-4.
18. Thein H, Yi Q, Dore G, Krahn M. Estimation of stage specific fibrosis progression rates in chronic hepatitis C virus infection: A meta analysis and meta regression. Hepatology. 2008;48(2):418-31.
19. Janjua NZ, Chong M, Kuo M, Woods R, Wong J, Yoshida EM, et al. Long-term effect of sustained virological response on hepatocellular carcinoma in patients with hepatitis C in Canada. Journal of hepatology. 2017;66(3):504-13.
20. Kanwal F, Kramer J, Asch SM, Chayanupatkul M, Cao Y, El-Serag HB. Risk of hepatocellular cancer in HCV patients treated with direct-acting antiviral agents. Gastroenterology. 2017;153(4):996-1005. e1.
21. Fattovich G, Giustina G, Degos F, Tremolada F, Diodati G, Almasio P, et al. Morbidity and mortality in compensated cirrhosis type C: a retrospective follow-up study of 384 patients. Gastroenterology. 1997;112(2):463-72.
22. Planas R, Ballesté B, Antonio Álvarez M, Rivera M, Montoliu S, Anton Galeras J, et al. Natural history of decompensated hepatitis C virus-related cirrhosis. A study of 200 patients. Journal of hepatology. 2004;40(5):823-30.

© 2020 Chen Q et al. *JAMA Network Open.*

23. Romano A, Angeli P, Piovesan S, Noventa F, Anastassopoulos G, Chemello L, et al. Newly diagnosed hepatocellular carcinoma in patients with advanced hepatitis C treated with DAAs: a prospective population study. Journal of hepatology. 2018;69(2):345-52.
24. Thuluvath P, Guidinger M, Fung J, Johnson L, Rayhill S, Pelletier S. Liver transplantation in the United States, 1999–2008. American Journal of Transplantation. 2010;10(4p2):1003-19.
25. Davis G, Alter M, El-Serag H, Poynard T, Jennings L. Aging of hepatitis C virus (HCV)-infected persons in the United States: a multiple cohort model of HCV prevalence and disease progression. Gastroenterology. 2010;138(2):513-21.
26. Lang K, Danchenko N, Gondek K, Shah S, Thompson D. The burden of illness associated with hepatocellular carcinoma in the United States. Journal of hepatology. 2009;50(1):89-99.
27. Saab S, Hunt DR, Stone MA, McClune A, Tong MJ. Timing of hepatitis C antiviral therapy in patients with advanced liver disease: A decision analysis model. Liver Transpl. 2010;16(6):748-59.
28. Wolfe R, Roys E, Merion R. Trends in Organ Donation and Transplantation in the United States, 1999–2008. American Journal of Transplantation. 2010;10(4p2):961-72.
29. Hep C State Policy Simulator 2019 [Available from: https://www.hepcsimulator.org/.
30. Shiffman ML, Suter F, Bacon BR, Nelson D, Harley H, Solá R, et al. Peginterferon Alfa-2a and Ribavirin for 16 or 24 Weeks in HCV Genotype 2 or 3. New England Journal of Medicine. 2007;357(2):124-34.
31. McHutchison JG, Lawitz EJ, Shiffman ML, Muir AJ, Galler GW, McCone J, et al. Peginterferon Alfa-2b or Alfa-2a with Ribavirin for Treatment of Hepatitis C Infection. New England Journal of Medicine. 2009;361(6):580-93.
32. Talal A, LaFleur J, Hoop R, Pandya P, Martin P, Jacobson I, et al. Absolute and relative contraindications to pegylated‐interferon or ribavirin in the US general patient population with chronic hepatitis C: results from a US database of over 45 000 HCV‐infected, evaluated patients. Alimentary pharmacology & therapeutics. 2013;37(4):473-81.
33. Shiffman ML, Di Bisceglie AM, Lindsay KL, Morishima C, Wright EC, Everson GT, et al. Peginterferon Alfa-2a and ribavirin in patients with chronic hepatitis C who have failed prior treatment1, 2, 3 1 2 3. Gastroenterology. 2004;126(4):1015-23.
34. Poynard T, Colombo M, Bruix J, Schiff E, Terg R, Flamm S, et al. Peginterferon alfa-2b and ribavirin: effective in patients with hepatitis C who failed interferon alfa/ribavirin therapy. Gastroenterology. 2009;136(5):1618-28.
35. Jacobson IM, McHutchison JG, Dusheiko G, Di Bisceglie AM, Reddy KR, Bzowej NH, et al. Telaprevir for previously untreated chronic hepatitis C virus infection. New England Journal of Medicine. 2011;364(25):2405-16.
36. Poordad F, McCone Jr J, Bacon BR, Bruno S, Manns MP, Sulkowski MS, et al. Boceprevir for untreated chronic HCV genotype 1 infection. New England Journal of Medicine. 2011;364(13):1195-206.
37. Bacon BR, Gordon SC, Lawitz E, Marcellin P, Vierling JM, Zeuzem S, et al. Boceprevir for previously treated chronic HCV genotype 1 infection. New England Journal of Medicine. 2011;364(13):1207-17.
38. Zeuzem S, Andreone P, Pol S, Lawitz E, Diago M, Roberts S, et al. Telaprevir for retreatment of HCV infection. New England Journal of Medicine. 2011;364(25):2417-28.
39. Lawitz E, Mangia A, Wyles D, Rodriguez-Torres M, Hassanein T, Gordon SC, et al. Sofosbuvir for previously untreated chronic hepatitis C infection. New England Journal of Medicine. 2013;368(20):1878-87.
40. Jacobson IM, Gordon SC, Kowdley KV, Yoshida EM, Rodriguez-Torres M, Sulkowski MS, et al. Sofosbuvir for hepatitis C genotype 2 or 3 in patients without treatment options. New England Journal of Medicine. 2013;368(20):1867-77.
41. Lawitz E, Sulkowski MS, Ghalib R, Rodriguez-Torres M, Younossi ZM, Corregidor A, et al. Simeprevir plus sofosbuvir, with or without ribavirin, to treat chronic infection with hepatitis C virus genotype 1 in non-responders to pegylated interferon and ribavirin and treatment-naive patients: the COSMOS randomised study. The Lancet. 2014;384(9956):1756-65.
42. Zeuzem S, Dusheiko GM, Salupere R, Mangia A, Flisiak R, Hyland RH, et al. Sofosbuvir and ribavirin in HCV genotypes 2 and 3. New England Journal of Medicine. 2014;370(21):1993-2001.
43. Kowdley KV, Gordon SC, Reddy KR, Rossaro L, Bernstein DE, Lawitz E, et al. Ledipasvir and sofosbuvir for 8 or 12 weeks for chronic HCV without cirrhosis. New England Journal of Medicine. 2014;370(20):1879-88.

© 2020 Chen Q et al. *JAMA Network Open.*

44. Sulkowski MS, Gardiner DF, Rodriguez-Torres M, Reddy KR, Hassanein T, Jacobson I, et al. Daclatasvir plus sofosbuvir for previously treated or untreated chronic HCV infection. New England Journal of Medicine. 2014;370(3):211-21.
45. Afdhal N, Reddy KR, Nelson DR, Lawitz E, Gordon SC, Schiff E, et al. Ledipasvir and sofosbuvir for previously treated HCV genotype 1 infection. The New England journal of medicine. 2014;370(16):1483-93.
46. Feld JJ, Kowdley KV, Coakley E, Sigal S, Nelson DR, Crawford D, et al. Treatment of HCV with ABT-450/r-ombitasvir and dasabuvir with ribavirin. The New England journal of medicine. 2014;370(17):1594-603.
47. Ferenci P, Bernstein D, Lalezari J, Cohen D, Luo Y, Cooper C, et al. ABT-450/r–Ombitasvir and Dasabuvir with or without Ribavirin for HCV. New England Journal of Medicine. 2014;370(21):1983-92.
48. Poordad F, Hezode C, Trinh R, Kowdley KV, Zeuzem S, Agarwal K, et al. ABT-450/r-ombitasvir and dasabuvir with ribavirin for hepatitis C with cirrhosis. The New England journal of medicine. 2014;370(21):1973-82.
49. Zeuzem S, Jacobson IM, Baykal T, Marinho RT, Poordad F, Bourliere M, et al. Retreatment of HCV with ABT-450/r-ombitasvir and dasabuvir with ribavirin. The New England journal of medicine. 2014;370(17):1604-14.
50. Flisiak R, Janczewska E, Wawrzynowicz-Syczewska M, Jaroszewicz J, Zarebska-Michaluk D, Nazzal K, et al. Real-world effectiveness and safety of ombitasvir/paritaprevir/ritonavir +/- dasabuvir +/- ribavirin in hepatitis C: AMBER study. Aliment Pharmacol Ther. 2016;44(9):946-56.
51. Feld JJ, Jacobson IM, Hézode C, Asselah T, Ruane PJ, Gruener N, et al. Sofosbuvir and Velpatasvir for HCV Genotype 1, 2, 4, 5, and 6 Infection. New England Journal of Medicine. 2015;373(27):2599-607.
52. Roth D, Nelson DR, Bruchfeld A, Liapakis A, Silva M, Monsour H, et al. Grazoprevir plus elbasvir in treatment-naive and treatment-experienced patients with hepatitis C virus genotype 1 infection and stage 4–5 chronic kidney disease (the C-SURFER study): a combination phase 3 study. The Lancet. 2015;386(10003):1537-45.
53. Forns X, Gordon SC, Zuckerman E, Lawitz E, Calleja JL, Hofer H, et al. Grazoprevir and elbasvir plus ribavirin for chronic HCV genotype-1 infection after failure of combination therapy containing a direct-acting antiviral agent. Journal of hepatology. 2015;63(3):564-72.
54. Burstow NJ, Mohamed Z, Gomaa AI, Sonderup MW, Cook NA, Waked I, et al. Hepatitis C treatment: where are we now? International Journal of General Medicine. 2017;10:39-52.
55. Bourlière M, Gordon SC, Flamm SL, Cooper CL, Ramji A, Tong M, et al. Sofosbuvir, Velpatasvir, and Voxilaprevir for Previously Treated HCV Infection. New England Journal of Medicine. 2017;376(22):2134-46.
56. Forns X, Lee SS, Valdes J, Lens S, Ghalib R, Aguilar H, et al. Glecaprevir plus pibrentasvir for chronic hepatitis C virus genotype 1, 2, 4, 5, or 6 infection in adults with compensated cirrhosis (EXPEDITION-1): a single-arm, open-label, multicentre phase 3 trial. The Lancet Infectious Diseases. 2017;17(10):1062-8.
57. Volk ML, Tocco R, Saini S, Lok ASF. Public health impact of antiviral therapy for hepatitis C in the United States. Hepatology. 2009;50(6):1750-5.
58. Silverman E. What the 'Shocking' Gilead Discounts on its Hepatitis C Drugs Will Mean. Wall Street Journal. February 4, 2015. Retrieved from http://blogs.wsj.com/pharmalot/2015/02/04/what-the-shocking-gilead-discounts-on-its-hepatitis-c-drugs-will-mean/ (last accessed: April 9, 2015). 2015.
59. Gilead Sciences Earnings Report: Q2 2015 Conference Call Transcript. Retrieved from: http://www.thestreet.com/story/13235916/1/gilead-sciences-gild-earnings-report-q2-2015-conference-call-transcript.html, last accessed: September 28, 2015). 2015.
60. Beckman AL, Bilinski A, Boyko R, Camp GM, Wall AT, Lim JK, et al. New Hepatitis C Drugs Are Very Costly And Unavailable To Many State Prisoners. Health Aff (Millwood). 2016;35(10):1893-901.
61. Denniston MM, Klevens RM, McQuillan GM, Jiles RB. Awareness of infection, knowledge of hepatitis C, and medical follow‐up among individuals testing positive for hepatitis C: National Health and Nutrition Examination Survey 2001‐2008. Hepatology. 2012;55(6):1652-61.
62. Stepanova M, Kanwal F, El‐Serag HB, Younossi ZM. Insurance status and treatment candidacy of hepatitis C patients: Analysis of population‐based data from the United States. Hepatology. 2011;53(3):737-45.

© 2020 Chen Q et al. *JAMA Network Open.*

63. Kates J. Medicaid coverage and care for the homeless population: key lessons to consider for the 2014 Medicaid Expansion: Henry J. Kaiser Family Foundation; 2012.
64. Congressional Budget Office. Updated Budget Projections: 2014 to 2024. Pub. No. 4928. April 2014 (retrieved from www.cbo.gov/publication/45229, last accessed: September 28, 2015). 2014.

© 2020 Chen Q et al. *JAMA Network Open*.
